# Multi-omics identifies PCK1-associated immunometabolic regulation underlying the protective effects of sinomenine in vascular dementia

**DOI:** 10.3389/fphar.2026.1979177

**Published:** 2026-10-29

**Authors:** Wanling Ren, Zipu Wang, Weisi Yang, Yuan Rao, Zhiqing Qiu, Jing Li, Haining Ou

**Affiliations:** 1 Traditional Chinese Medicine Rehabilitation Research Team, The Second Affiliated Hospital of Guangzhou University of Chinese Medicine, Guangzhou, China; 2 School of Pharmaceutical Sciences, Guangzhou University of Chinese Medicine, Guangzhou, China; 3 The Fifth Affiliated Hospital of Guangzhou Medical University, Guangzhou, China; 4 Rehabilitation Department, The Second Affiliated Hospital of Guangzhou University of Chinese Medicine, Guangzhou, China; 5 Guangdong Provincial Academy of Chinese Medical Sciences, Guangdong Provincial Hospital of Chinese Medicine, Guangzhou, China

**Keywords:** metabolic reprogramming, microglia, neuroinflammation, PCK1, single-nucleus RNA sequencing, sinomenine, vascular dementia

## Abstract

**Background:**

Vascular dementia (VaD) is one of the major causes of cognitive impairment among older adults. However, effective treatments that can modify the progression of the disease are still lacking. Emerging evidence indicates that metabolic remodeling in microglia influences inflammatory responses during vascular brain injury. Microglial immunometabolic regulation has therefore been proposed as an important contributor to the pathogenesis of vascular dementia (VaD).

**Methods:**

Transcriptomic integration, WGCNA, network pharmacology, and single-nucleus RNA sequencing were combined to identify disease-associated microglial alterations and potential targets involved in sinomenine (SIN)-mediated regulation. The interaction between SIN and phosphoenolpyruvate carboxykinase 1 (PCK1) was investigated using molecular docking and molecular dynamics simulations. The biological effects of SIN were then validated in lipopolysaccharide (LPS)-stimulated BV2 microglia.

**Results:**

Integrated analyses highlighted PCK1 as a potential mediator associated with SIN-induced metabolic regulation in VaD. snRNA-seq demonstrated expansion of metabolically stressed microglial states accompanied by PCK1-related metabolic alterations. Stable binding between SIN and PCK1 was supported by molecular docking and molecular dynamics simulations. In LPS-stimulated BV2 microglia, SIN suppressed inflammatory activation, restored PCK1 expression, attenuated inflammation-associated metabolic remodeling, improved cellular bioenergetics, and modulated metabolic redox-related parameters. SIN also enhanced adaptive stress responses through regulation of CRYAB, APOD, and HSPH1.

**Conclusion:**

Collectively, these findings suggest that SIN alleviates VaD-associated microglial dysfunction by modulating PCK1-associated immunometabolic alterations. They expand the current understanding of the pharmacological actions of SIN and highlight microglial immunometabolic regulation as a potential therapeutic target for VaD.

## 1 Introduction

Vascular dementia (VaD) is the second most common form of dementia and poses a significant clinical challenge worldwide. VaD involves progressive cognitive impairment resulting from cerebrovascular damage, long-term cerebral hypoperfusion, and disruption of neurovascular homeostasis (Iadecola et al., 2019; Rundek et al., 2022; Rajeev et al., 2023). Although current therapeutic strategies mainly focus on controlling vascular risk factors, effective disease-modifying treatments for VaD remain limited (Mok et al., 2024). Recent studies have shown that ongoing neuroinflammation, oxidative stress, and impaired immune function contribute to the development of VaD (Tian et al., 2022; Yang et al., 2022), highlighting the need to identify novel molecular mechanisms and therapeutic targets.

As the main innate immune cells in the brain, microglia constantly monitor the neural environment and respond to vascular and metabolic changes (Zeng et al., 2024). After cerebrovascular injury, excessive activation of microglia increases the release of pro-inflammatory factors, such as inducible nitric oxide synthase (iNOS), tumor necrosis factor-α (TNF-α), and interleukin-1β (IL-1β) (Shi and Yong, 2025; Tack et al., 2025). These inflammatory mediators further worsen neuronal damage and cognitive impairment. Recent advances in single-cell and single-nucleus transcriptomic analyses have shown that microglial functional states are strongly associated with their metabolic profiles (Hammond et al., 2019; Olah et al., 2020). These metabolic programs involve glycolysis, lactate metabolism, mitochondrial oxidative phosphorylation, and redox regulation (Rana et al., 2025; Asimakidou et al., 2025; Jung et al., 2025). Therefore, immunometabolic remodeling may serve as a key mechanism that maintains chronic microglial activation during VaD progression.

Among metabolic regulators, enzymes controlling carbon flux may represent critical determinants of microglial functional states because metabolic intermediates directly influence inflammatory signaling. Phosphoenolpyruvate carboxykinase 1 (PCK1) is a cytosolic phosphoenolpyruvate carboxykinase that converts oxaloacetate into phosphoenolpyruvate. Through this process, PCK1 affects intracellular carbon distribution and helps maintain metabolic flexibility. PCK1 was traditionally investigated for its role in hepatic glucose metabolism. However, recent studies have shown that it also participates in immune-cell metabolic regulation and stress responses (Bernier et al., 2020; Cheng et al., 2021; Lauro and Limatola, 2020). However, the role of PCK1 in microglial immunometabolic remodeling and its contribution to VaD-related neuroinflammation have not yet been fully clarified.

Sinomenine (SIN) is a naturally derived isoquinoline alkaloid extracted from *Sinomenium acutum* and has attracted extensive attention due to its diverse pharmacological effects (Zhen et al., 2026). Previous studies have shown that SIN can regulate inflammatory responses and oxidative stress in various neurological disease models, including cerebral ischemia, Alzheimer’s disease, and Parkinson’s disease (Hong et al., 2022). Mechanistically, SIN has been reported to modulate inflammatory signaling pathways, such as NF-κB activation and NLRP3 inflammasome-related pathways (Qiu et al., 2016). These effects may help reduce excessive glial activation and alleviate inflammation-induced neuronal damage, whether SIN modulates microglial immunometabolism remains unknown.

Here, we integrated transcriptomic analysis, network pharmacology, single-nucleus RNA sequencing (snRNA-seq), and cellular experiments to characterize the mechanism of SIN-mediated protection in VaD. We identified PCK1 as a potential metabolic candidate associated with SIN-mediated protection and revealed stress-response microglial states in VaD. Furthermore, we demonstrated that SIN alleviated inflammatory activation and restored metabolic dysfunction in LPS-stimulated BV2 microglia. Together, these data suggest that modulation of PCK1-associated metabolic pathways may influence microglial inflammatory and metabolic states in VaD.

## 2 Materials and methods

### 2.1 Data acquisition and preprocessing

We obtained transcriptomic datasets for vascular dementia (VaD) from the GEO database. The microarray expression matrix of dataset GSE122063 was annotated based on the GPL16699 platform information, which included frontal and temporal cortex tissues from 8 VaD patients and 11 non-demented healthy controls. The snRNA-seq dataset GSE282111 was annotated based on the platform information from GPL24676, comprising periventricular white matter tissues adjacent to the frontal horn from 4 VaD patients and four non-demented healthy controls.

### 2.2 Differential expression analysis

We performed differential expression analysis on the GSE122063 dataset using the limma package in R software (version 4.2.1) to identify differentially expressed genes (DEGs). DEGs were selected based on the thresholds of |log_2_ fold change (logFC)| > 0.585 and adjusted P < 0.05. The identified DEGs were further visualized through volcano plots and heatmaps produced with the ggplot2 and pheatmap packages.

### 2.3 GO/KEGG enrichment

Further functional annotation analysis was carried out using clusterProfiler. Human gene annotations were mapped to Entrez IDs through the org.Hs.e.g.,.db package. GO enrichment analysis and KEGG pathway analysis were then performed with an FDR cutoff of <0.05.

### 2.4 GSEA

Gene set enrichment analysis (GSEA) was applied to explore the biological processes and signaling pathways related to PCK1 expression. All detected genes were ordered according to the Spearman correlation values between PCK1 expression and individual gene expression levels. The generated ranked gene list was subsequently analyzed for pathway enrichment. The Hallmark gene sets obtained from the Molecular Signatures Database were selected to assess pathway activity. Significance evaluation was performed with 1,000 permutation tests, and the Benjamini–Hochberg method was applied for multiple testing correction. Gene sets with an FDR value below 0.05 were regarded as significantly enriched.

### 2.5 WGCNA analysis

Weighted gene co-expression network analysis (WGCNA) was conducted with the WGCNA R package to identify gene modules related to VaD. The genes showing the highest expression variation (top 25%) were selected for network analysis. The soft-thresholding power was set at β = 2. The minimum number of genes in each module was set to 120, and the module merging threshold was set at 0.25. Module eigengenes were then compared with disease status (VaD vs. Control) using Pearson correlation analysis. Hub genes were selected based on intramodular connectivity and module membership (MM). Finally, the DEGs (|log_2_FC| > 0.585, adjusted p < 0.05) were compared with the WGCNA modules to identify candidate genes associated with VaD.

### 2.6 Molecular docking and molecular dynamics simulation

The 2D structure of the small molecule was obtained from the PubChem database and converted into a three-dimensional structure using ChemOffice. The optimized ligand structure was saved in MOL2 format for docking analysis. The crystal structure of the target protein was downloaded from the RCSB Protein Data Bank (PDB), and a high-resolution structure was selected as the receptor. Protein preparation, including removal of water molecules and unnecessary heteroatoms, was performed using PyMOL 2.6. AutoDockTools 1.5.6 was applied for receptor and ligand preparation, including hydrogen addition and rotatable bond assignment. Molecular docking was carried out using AutoDock Vina, and the optimal binding conformation was selected based on docking affinity. The binding modes and intermolecular interactions were further analyzed using Discovery Studio 2019 and PyMOL 2.6.

Molecular dynamics simulations were performed using GROMACS 2022.2. The protein and ligand were parameterized using the AMBER14SB and GAFF2 force fields, respectively, with ligand charges calculated by the RESP method. The complex system was solvated using the TIP3P water model in a cubic box with a 1 nm buffer distance, and Na^+^/Cl^−^ ions were added to maintain electroneutrality and physiological ionic strength (0.15 M). Following energy minimization and NVT/NPT equilibration, a 100 ns production simulation was performed at 310 K and 1 bar with a 2 fs time step. RMSD, RMSF, radius of gyration (Rg), SASA, hydrogen bonds, and free energy landscape analyses were conducted using GROMACS tools to evaluate the stability and conformational dynamics of the protein–ligand complex.

### 2.7 SnRNA-seq data processing and analysis

SnRNA-seq data analysis was performed using the Seurat R package. Low-quality nuclei were removed during quality control, including nuclei with fewer than 200 detected genes, mitochondrial gene content >10%, detected genes <300 or >10,000, or unique molecular identifiers (UMIs) < 200. Genes detected in fewer than three nuclei were also excluded. After quality control, the expression matrices were normalized using the NormalizeData function. Highly variable genes were identified using the FindVariableFeatures function with the variance-stabilizing transformation (vst) method, and the top 2,000 genes were selected for subsequent analysis. Data scaling was performed by regressing out nCount_RNA and mitochondrial gene percentage. Batch effects were corrected using Harmony integration with sample identity (orig.ident) as the grouping variable. The first 20 principal components were used for integration, followed by dimensionality reduction using PCA, UMAP, and t-SNE. Graph-based clustering was performed with resolution values ranging from 0.1 to 1.0.

Microglial clusters were further annotated according to their transcriptional signatures by integrating marker gene expression patterns with previously reported single-cell microglial state classifications. Phagocytic microglia were defined by enrichment of phagocytosis- and lysosome-associated genes, including FOLR2, MRC1, CD68, and MSR1 (Hammond et al., 2019). Antigen-presenting microglial populations showed increased expression of MHC-II-associated markers, including HLA-DRA, HLA-DRB1, CD74, and CIITA, consistent with their antigen processing and presentation functions (Mathys et al., 2017; Olah et al., 2020). MHC-II transitional microglia were annotated according to the expression patterns of immune transition and metabolic adaptation markers, including CD83, SYTL3, and PFKFB4. Interferon-response microglia were annotated according to interferon-stimulated gene signatures, including ISG15, IFITM3, MX1, STAT1, and IRF7 (Hammond et al., 2019). Stress-response microglia were identified based on transcriptional signatures associated with cellular stress responses, lipid metabolism, and adaptive processes, including HSPH1, HSPA4, CRYAB, and APOD (Corraliza-Gomez et al., 2023; Guo et al., 2019; Krasemann et al., 2017; van den Brink et al., 2017).

PCK1-associated metabolic gene signatures were generated based on genes involved in pathways enriched in PCK1-associated analysis, including glycolysis, pyruvate metabolism, gluconeogenesis, oxidative phosphorylation, lipid metabolism, and energy metabolism.

### 2.8 Cell experiment reagents

BV2 microglial cells were supplied by Procell Life Science and Technology Co., Ltd. (Wuhan, China). The cells were grown in high-glucose DMEM (PM150210, Procell) supplemented with 10% fetal bovine serum and 1% penicillin–streptomycin. Cell cultures were maintained at 37 °C in a humidified incubator with 5% CO_2_. When cell confluence reached approximately 70%–80%, the experiments were conducted. Each *in vitro* assay was repeated with at least three independent biological replicates.

Sinomenine hydrochloride (purity >98%, S837339, Macklin) was first dissolved in DMSO to generate a 100 mmol/L stock solution. LPS (L2880, Sigma-Aldrich) was prepared in PBS at 1.5 mg/mL. The required working concentrations were freshly prepared through dilution of the stock solutions in complete medium before cell treatment. The DMSO content was maintained at the same level among all treatment groups and vehicle controls, with a final concentration not exceeding 0.1% (v/v).

### 2.9 Cell viability

For cell viability assays, BV2 cells were plated in 96-well plates at a density of 5 × 10^3^ cells/well and cultured for 24 h. The cells were then exposed to different concentrations of sinomenine (0, 25, 50, 100, 125, 150, 175, and 200 μM) for 24 h or treated with LPS (0, 125, 250, 500, and 1000 ng/mL) for 4 h. Each group was prepared with at least five technical replicates. After treatment, cell viability was assessed using the CCK-8 assay. Each well received 10 μL of CCK-8 reagent (GK10001, GLPBIO), followed by incubation at 37 °C for 1 h in the dark. The optical density was subsequently recorded at 450 nm using a microplate reader. Cell viability was calculated by comparing the absorbance values with those of the untreated control group and was presented as a relative percentage.

### 2.10 LPS-induced inflammatory model in BV2 microglial cells and sinomenine intervention

Cells were seeded into 6-well plates and treated when the cell confluence reached 60%–70%. Different concentrations of sinomenine (25, 50 and 100 μM) were added for 24 h before LPS exposure (250 ng/mL, 4 h). Following treatment, cell pellets and supernatants were harvested separately for further analysis.

### 2.11 qRT-PCR analysis

Total RNA was isolated from BV2 cells with TRIzol reagent. The obtained RNA was subsequently reverse-transcribed into cDNA with a reverse transcription kit (AG11728, Accurate Biotechnology, Changsha, China). Quantitative PCR was performed using SYBR Green qPCR Master Mix (AG11735, Accurate Biotechnology, Changsha, China) on a Bio-Rad CFX96 system. β-actin was selected as the reference gene, and relative mRNA expression was calculated using the 2^−ΔΔCt method. Primer information is summarized in Table 1.

**TABLE 1:** Primer sequences.

Gene	Forward primer	Reverse primer
Nos2	ACA​TGC​AAG​GAA​GGG​AAC​TCT	GGA​CGA​GAC​GGA​TAG​GCA​GA
Il1b	ACT​CCA​CTT​TGC​TCT​TGA​CTT​CT	AAT​GAA​AGA​CGG​CAC​ACC​CA
Il10	GAT​AAG​GCT​TGG​CAA​CCC​AAG​TAA	GCC​AGA​GCC​ACA​TGC​TCC​TA
Mrc1	AGA​GCT​GGC​GAG​CAT​CAA​GAG	TTC​CAT​AGG​TCA​GTC​CCA​ACC​AA
Hk2	CGT​GGA​TGG​CTC​TGT​CTA​CAA​G	AGG​AAG​CGG​ACA​TCA​CAA​TCG
Glut1	GTT​TGT​TGT​AGA​GCG​AGC​TGG​A	CTC​AGA​TAG​GAC​ATC​CAA​GGC​AG
Pck1	CCG​AAG​TTG​TAG​CCG​AAG​AAG	TTG​TAG​GAG​CAG​CCA​TGA​GAT
Cryab	GCC​TCT​TCG​ACC​AGT​TCT​TC	AAC​GCA​TCT​CTG​AGA​GTC​CG
Apod	CCA​CCG​GCA​CCC​TAC​TGG​ATC	CGG​GCA​GTT​CGC​TTG​ATC​TGT
Hsph1	TCT​TCA​GTG​TGG​AGC​AGA​TAA​C	GAA​GAA​GGA​TGG​GAC​TGA​GAT​G
Actb	CAT​CCG​TAA​AGA​CCT​CTA​TGC​CAA​C	ATG​GAG​CCA​CCG​ATC​CAC​A

### 2.12 Western blot analysis

BV2 cells were lysed in RIPA buffer containing protease and phosphatase inhibitors. Protein concentrations were determined using a BCA assay kit. Equal amounts of protein (40 μg) were separated by SDS-PAGE and transferred to PVDF membranes. The membranes were blocked with 5% non-fat milk and incubated with primary antibodies against HK2 (1:1,000, ab209847, Abcam), PCK1 (1:1,000, 12940T, Cell Signaling Technology), and β-actin (1:1,000, GB15003, Servicebio) overnight at 4 °C. After incubation with HRP-conjugated anti-rabbit IgG secondary antibody (1:5,000, GB152199, Servicebio), protein signals were detected using an ECL substrate kit (G2014, Servicebio).

### 2.13 Enzyme-linked immunosorbent assay (ELISA)

The concentrations of inflammatory cytokines in the culture supernatants were evaluated by ELISA. After different treatments, cell supernatants were harvested and used to detect TNF-α, IL-1β, IL-10, and TGF-β with commercial ELISA kits (Neobioscience). All procedures were performed following the manufacturer’s instructions. The absorbance values were measured at 450 nm using a microplate reader, and cytokine levels were calculated from the generated standard curves.

### 2.14 Immunofluorescence staining

Immunofluorescence analysis was conducted to assess microglial inflammatory phenotype by examining iNOS and CD206 expression. BV2 cells were grown on coverslips in 24-well plates and treated as required. After treatment, cells were fixed with cold methanol at −20 °C for 15 min. The fixed cells were then permeabilized with 0.1% Triton X-100 and blocked with 5% BSA for 1 h. Cells were incubated with primary antibodies targeting iNOS (1:400, #13120, Cell Signaling Technology) and CD206 (1:100, MA516871, Invitrogen) at 4 °C overnight. After incubation with fluorescent secondary antibodies (1:500, 32731/A-21434, Invitrogen) for 1.5 h at room temperature in the dark, nuclei were counterstained with DAPI (C1006, Beyotime). Fluorescence signals were visualized using a spinning-disk confocal microscope, and representative images were collected for analysis.

### 2.15 Metabolic and redox analysis

Cellular metabolic and redox parameters were analyzed using commercial assay kits. Lactate production was assessed in culture supernatants using an L-lactate assay kit (S0227S, Beyotime), and absorbance was measured at 530–570 nm. Cellular ATP levels were determined with a luciferase-based ATP assay kit (S0026, Beyotime) after cell lysis, and luminescence signals were recorded. The NAD^+^/NADH ratio was measured with an NAD^+^/NADH assay kit (S0175, Beyotime) based on the measurement of total NAD and NADH levels after selective NAD^+^ removal. Intracellular ROS accumulation was detected by DCFH-DA staining (S0033S, Beyotime). Briefly, cells were treated with 10 μM DCFH-DA for 30 min in the dark, and fluorescence signals were subsequently recorded. The obtained metabolic data were adjusted according to total protein content when necessary.

### 2.16 Statistical analysis

All quantitative data were obtained from at least three independent experiments. Statistical analyses were conducted using GraphPad Prism 9.3.0 (GraphPad Software, USA), and data are presented as mean ± SD. The Shapiro–Wilk test was used to assess normality, while the Brown–Forsythe test was employed to evaluate homogeneity of variances. For data meeting the assumptions of normality and equal variance, one-way ANOVA followed by Bonferroni’s multiple comparisons test was performed. Welch’s ANOVA with the Games–Howell test was applied when variance homogeneity was not satisfied. Nonparametric data were analyzed using the Kruskal–Wallis test with Dunn’s multiple comparisons. A two-tailed p value < 0.05 was considered statistically significant. Western blot band intensity and immunofluorescence signal quantification were analyzed using ImageJ software (version 1.53).

## 3 Results

### 3.1 Screening of VaD-related genes and identification of disease-associated modules

To characterize VaD-associated transcriptional alterations, differential expression analysis was conducted using the GSE122063 dataset. A total of 293 DEGs were identified between VaD and control samples (Figures 1A,B). WGCNA analysis was then performed to investigate gene co-expression patterns and identify disease-related modules. Among the identified modules, the purple module showed the highest positive association with VaD status (r = 0.47, P = 1 × 10^−5^) and included 281 genes (Figures 1C,E). Furthermore, genes in this module showed a strong relationship between gene significance and module membership (r = 0.79, P < 1 × 10^−61^). These results indicated that the purple module was closely linked to VaD-related molecular changes (Figure 1F). By intersecting the 281 genes in the WGCNA purple module with the 293 DEGs, 59 overlapping genes were identified (Figure 1D). The overlapping genes were regarded as potential VaD-related candidates and further analyzed through functional enrichment approaches.

**FIGURE 1:**
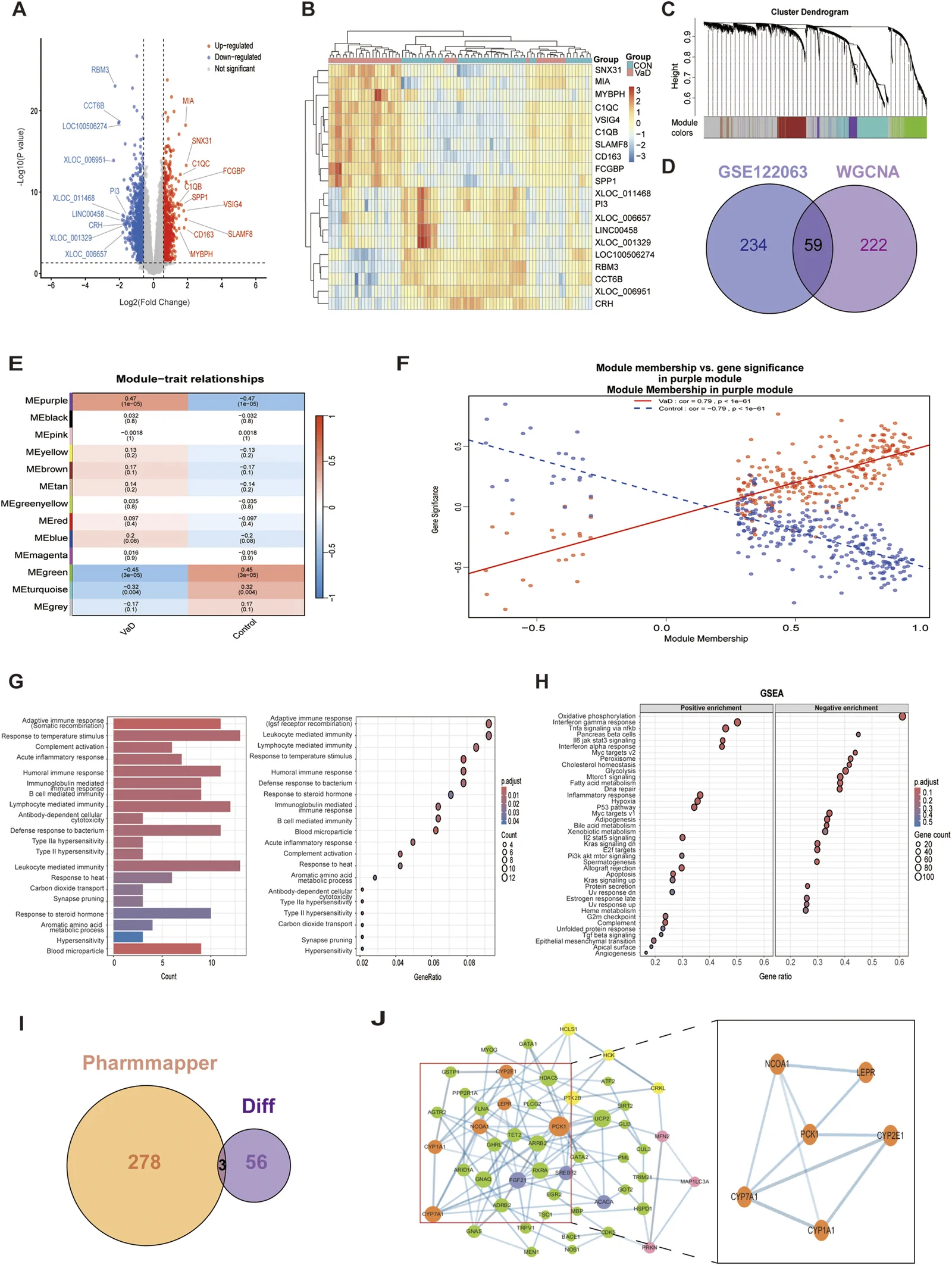
Identification of VaD-associated molecular signatures and potential therapeutic targets of sinomenine. **(A,B)** Volcano plot **(A)** and heatmap **(B)** showing DEGs between VaD and control samples. **(C)** WGCNA for identification of co-expressed modules. **(D)** Venn diagram illustrating the intersection of DEGs and genes within the WGCNA purple module. **(E)** Heatmap of module–trait correlations highlighting the module most strongly correlated with VaD. **(F)** Correlation between module membership and gene significance within the purple module. **(G)** GO enrichment analysis of VaD-associated candidate genes. **(H)** GSEA identifying significantly enriched pathways in VaD. **(I)** Venn diagram illustrating the overlap between predicted SIN targets and VaD-related genes. **(J)** PPI network highlighting hub genes among overlapping targets.

### 3.2 VaD is driven by coordinated immune activation and metabolic reprogramming

Enrichment profiling indicated that VaD-related genes were distributed across immune regulatory and metabolic pathways. Immune-associated terms, including complement activation, leukocyte-mediated immunity, and antigen presentation, were prominently represented, while glycolysis, oxidative phosphorylation, and MAPK signaling emerged as major metabolic pathways, indicating coupled immune-metabolic dysregulation in VaD (Figure 1G).

GSEA analysis further supported these findings. The activated microglial population exhibited stronger immune activity than other cell states. Pathway analysis revealed significant enrichment of interferon signaling, TNFA/NF-κB signaling, IL6/JAK/STAT3 signaling, inflammatory response, and complement pathways. In addition, apoptosis, p53 pathway, hypoxia, glycolysis, oxidative phosphorylation, and mTOR-related signaling were also enriched, indicating that immune activation was accompanied by cellular stress and metabolic remodeling (Figure 1H).

### 3.3 Identification of PCK1 as a candidate metabolic factor associated with the effects of sinomenine in VaD

Using the PharmMapper database, we predicted 281 potential pharmacological targets of sinomenine. These targets were then intersected with vascular dementia (VaD)-related genes, yielding three overlapping candidates: CFD, RNASE3, and PCK1 (Figure 1I). To further refine the candidate list, we retrieved VaD pathogenic genes from the GeneCards database and subjected the three intersecting genes alongside these pathogenic genes to protein–protein interaction (PPI) network analysis combined with MCODE clustering. This approach identified a core functional module comprising six nodes and ten edges, in which PCK1 was pinpointed as the hub gene (Figure 1J). Together, these results indicate that PCK1 may represent a candidate molecular link connecting VaD-associated metabolic alterations with the pharmacological effects of sinomenine.

### 3.4 PCK1 is related to the inflammation-metabolic reprogramming of VaD

Single-gene GSEA further revealed a close relationship between PCK1 expression and inflammatory signaling. Elevated PCK1 expression was accompanied by increased enrichment of TNFα/NF-κB signaling, IL6/JAK/STAT3 signaling, and interferon response pathways. In contrast, mitochondrial oxidative phosphorylation, lipid metabolism, and DNA repair pathways were negatively correlated with PCK1 expression, indicating a shift toward inflammatory and stress-related states (Figures 2A,B).

**FIGURE 2:**
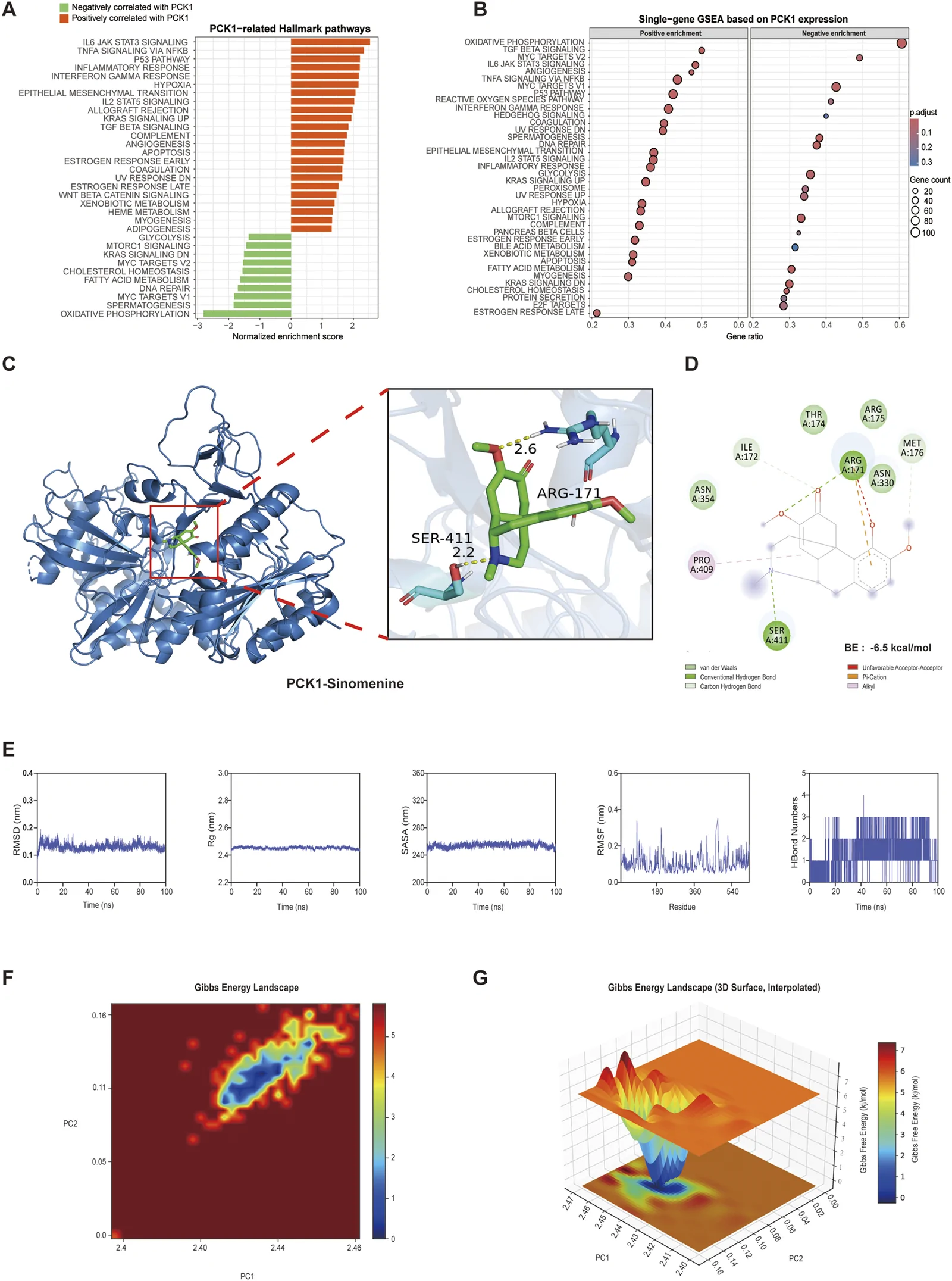
PCK1-associated pathway signatures and molecular interaction analysis of the PCK1–sinomenine complex. **(A)** Hallmark pathway enrichment analysis of PCK1-associated genes. **(B)** Single-gene GSEA based on PCK1 expression. **(C)** Three-dimensional docking model showing the interaction between sinomenine and PCK1. **(D)** Two-dimensional interaction profile of PCK1–SIN binding. **(E)** Molecular dynamics characterization of the PCK1–SIN complex, including RMSD, RMSF, Rg, SASA, and hydrogen-bond analyses. **(F,G)** Two- and three-dimensional Gibbs free energy landscapes of the PCK1–SIN complex.

### 3.5 Sinomenine is predicted to bind stably to PCK1

The molecular docking results suggested a favorable interaction between sinomenine and PCK1. The predicted binding affinity reached −6.5 kcal/mol, indicating stable binding. Structural modeling further demonstrated that hydrogen bonds were formed between sinomenine and the SER411 and ARG171 residues of PCK1, with bond distances of 2.2 Å and 2.6 Å, respectively. In addition, sinomenine interacted with PRO409 through hydrophobic interaction, with ASN354, THR174, ARG175, and ASN330 through van der Waals forces, with ILE172 and MET176 through carbon-hydrogen bonds, and with ARG171 through Pi-cation interaction, suggesting a favorable interaction network between sinomenine and PCK1 (Figures 2C,D).

The dynamic stability of the PCK1–sinomenine complex was investigated through molecular dynamics simulation. The results of RMSD, Rg, SASA, and RMSF showed that the complex maintained a stable overall conformation during the simulation, with only minor fluctuations in structural deviation, compactness, solvent-accessible surface area, and residue flexibility. Meanwhile, the number of hydrogen bonds between PCK1 and sinomenine was mainly maintained within the range of 0–4, indicating persistent interactions between the ligand and receptor (Figure 2E). Analysis of the Gibbs free energy landscape identified a concentrated low-energy region. This result further supported the conformational stability of the PCK1–sinomenine complex during binding. Collectively, these results indicate that sinomenine exhibits favorable binding affinity and dynamic stability with PCK1.

### 3.6 Microglial heterogeneity and PCK1-related metabolic remodeling in VaD

To analyze the cellular characteristics of vascular dementia (VaD), we analyzed the GSE282111 snRNA-seq dataset. UMAP analysis separated the nuclei into 11 transcriptionally distinct clusters. Cell identities were assigned according to established marker genes, allowing classification of seven major brain cell populations, including astrocytes, endothelial cells, microglia/macrophages, neurons, oligodendrocytes, and oligodendrocyte precursor cells, and a small population of uncharacterized cells (Figures 3A,B). Cell composition analysis showed that the overall proportions of major cell types were relatively comparable between VaD and control samples, suggesting that VaD-associated alterations may primarily involve cell-state remodeling rather than broad changes in major cell-type abundance (Figure 3C).

**FIGURE 3:**
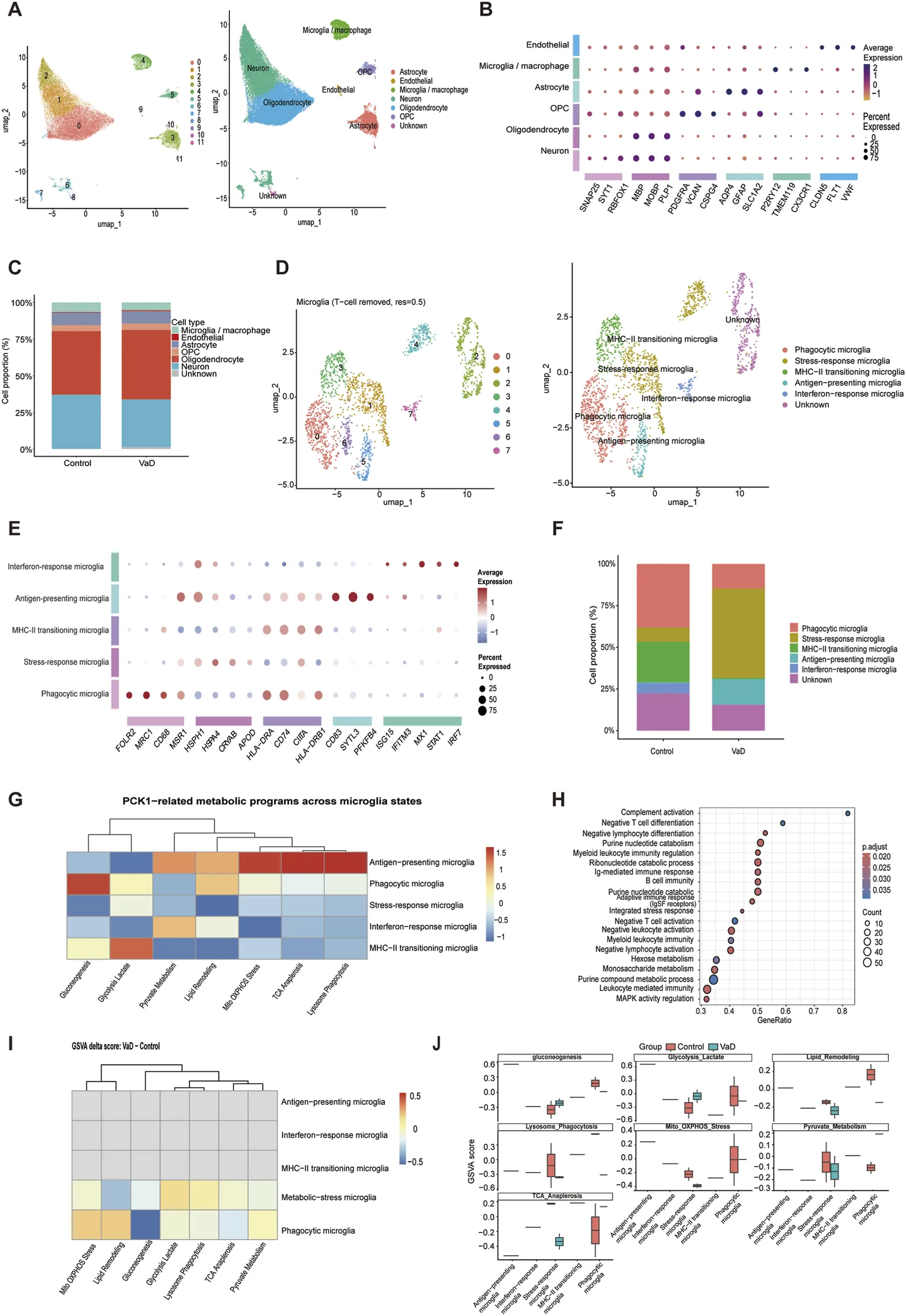
Single-nucleus transcriptomic analysis identifies stress-response microglial states and PCK1-related metabolic alterations in vascular dementia. **(A,B)** UMAP clustering **(A)** and canonical marker gene expression analysis **(B)** for annotation of major brain cell populations. **(C)** Comparison of major cell-type proportions between control and VaD samples. **(D,E)** Reclustering of microglia/myeloid cells **(D)** and marker gene expression analysis **(E)** identifying distinct microglial functional states. **(F)** Distribution and proportion changes of microglial states between the control and VaD groups. **(G)** Heatmap showing PCK1-associated metabolic program scores across different microglial states. **(H)** GO enrichment analysis of genes associated with underlying microglial state transitions. **(I)** GSVA delta-score heatmap showing VaD-associated pathway changes across different microglial states. **(J)** Pseudobulk GSVA analysis comparing PCK1-associated metabolic pathway changes between control and VaD groups.

To further dissect microglial heterogeneity, given their central roles in neuroinflammation, phagocytic clearance, and microenvironmental homeostasis, we performed subclustering analysis of the microglial population. Microglia were reclustered at a resolution of 0.5, yielding eight subclusters. Microglial subclusters were classified according to their marker gene profiles and associated transcriptional programs. Five major functional states were identified, including phagocytic, stress-response, MHC-II transitional, antigen-presenting, and interferon-responsive microglia, together with a minor uncharacterized population. DotPlot analysis demonstrated distinct marker gene expression patterns across these annotated states. Specifically, phagocytic microglia were characterized by high expression of FOLR2, MRC1, CD68, and MSR1; stress-response microglia preferentially expressed stress-associated genes including HSPH1, HSPA4, CRYAB, and APOD; MHC-II transitioning and antigen-presenting microglia showed enrichment of antigen presentation-related genes (HLA-DRA, CD74, and CIITA), whereas interferon-response microglia exhibited elevated expression of interferon-stimulated genes, including ISG15, IFITM3, MX1, STAT1, and IRF7 (Figure 3E).

Although total microglial abundance was not markedly altered, VaD samples exhibited clear shifts in microglial state composition. In particular, stress-response and antigen-presenting microglia were increased, whereas phagocytic and MHC-II transitional states were reduced, indicating disease-associated functional remodeling of microglia (Figure 3F). Because PCK1 transcripts are prone to technical dropout and low detection in snRNA-seq data, we used PCK1-related metabolic programs as functional surrogates to evaluate PCK1-associated activity across microglial states. This analysis revealed pronounced metabolic heterogeneity. Antigen-presenting microglia showed the highest overall activity of PCK1-related metabolic programs, including glycolysis, pyruvate metabolism, gluconeogenesis, lipid remodeling, and energy metabolism. In contrast, phagocytic microglia were enriched in lysosome-phagocytosis programs but showed relatively lower glucose metabolic activity, while stress-response microglia displayed broadly reduced metabolic pathway activity. MHC-II transitional and interferon-responsive microglia exhibited intermediate or selective pathway activation patterns (Figure 3G).

GSEA analysis of microglial DEGs revealed coordinated alterations in immune and metabolic programs. Immune-associated pathways, including pathways governing complement activation, leukocyte-mediated immunity, antigen processing and presentation, and lymphocyte activation, were prominently represented, along with metabolic pathways related to glucose metabolism, purine metabolism, and MAPK signaling regulation (Figure 3H). GSVA further supported state-dependent functional divergence among microglia, with distinct activity patterns across cell cycle, DAM, homeostatic, interferon-response, inflammatory, and MHC-related modules. Pseudobulk GSVA analysis confirmed that VaD-associated metabolic alterations were state-specific: stress-response microglia showed increased glycolysis-lactate and gluconeogenesis scores but reduced OXPHOS and lipid remodeling, phagocytic microglia showed decreased lysosome-phagocytosis activity, and antigen-presenting microglia displayed globally enhanced metabolic activity (Figures 3I,J). Collectively, these results indicate that VaD is characterized by state-dependent microglial immunometabolic remodeling, with PCK1-related metabolic programs closely associated with disease-linked microglial activation.

### 3.7 Sinomenine restores inflammatory-metabolic balance in activated microglia

Based on the bioinformatics analysis and molecular docking results, PCK1 was identified as a candidate metabolic regulator associated with the protective effects of sinomenine on microglial dysfunction. Among the microglial subpopulations identified by snRNA-seq, stress-response microglia exhibited prominent alterations in glycolytic activity and mitochondrial metabolism, which resembled the metabolic characteristics observed in LPS-stimulated BV2 cells. Therefore, an LPS-induced BV2 microglial model was used to examine whether SIN could alleviate inflammation-associated metabolic alterations.

#### 3.7.1 Sinomenine attenuates inflammatory activation and restores microglial homeostasis

Before functional experiments, the cytotoxic effects of SIN were evaluated by CCK-8 assay. BV2 cells were treated with increasing concentrations of SIN, followed by assessment of cell viability. While low concentrations of SIN had negligible effects on cell viability, higher concentrations caused a dose-dependent decline (Figure 4A). Therefore, 25, 50, and 100 μM SIN were selected for further experiments.

**FIGURE 4:**
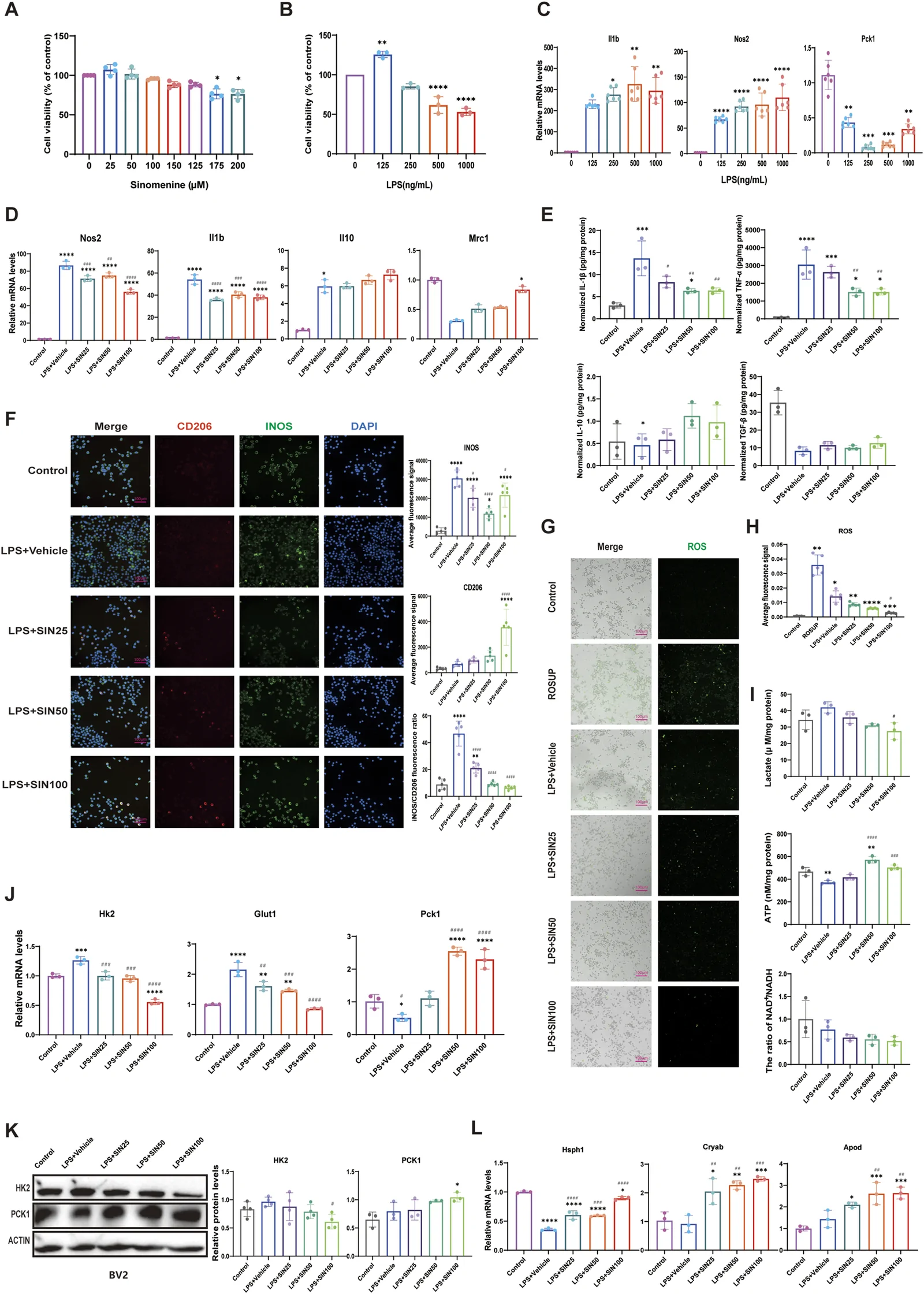
Sinomenine alleviates LPS-induced inflammatory activation and metabolic reprogramming in BV2 microglia. **(A,B)** CCK-8 analysis of BV2 cell viability following treatment with different concentrations of SIN **(A)** or LPS **(B)**. **(C)** qRT-PCR analysis of inflammation- and metabolism-related gene expression following SIN treatment alone. **(D)** qRT-PCR analysis of *Nos2*, *Il1b*, *Il10*, and *Mrc1* expression in LPS-stimulated BV2 cells following SIN pretreatment. **(E)** ELISA analysis of IL-1β, TNF-α, IL-10, and TGF-β production in LPS-stimulated BV2 cells following SIN pretreatment. **(F)** Representative immunofluorescence images and quantification of iNOS and CD206 expression in BV2 cells following SIN pretreatment. The iNOS/CD206 fluorescence ratio was used to assess microglial polarization. **(G)** Representative fluorescence images of intracellular ROS in BV2 cells following SIN pretreatment. **(H)** Quantification of intracellular ROS fluorescence intensity. **(I)** Effects of SIN pretreatment on glycolytic metabolism, including lactate production, ATP generation, and the NAD^+^/NADH ratio. **(J)** qRT-PCR analysis of *Hk2*, *Glut1*, and *Pck1* expression in LPS-stimulated BV2 cells following SIN pretreatment. **(K)** Western blot analysis and quantification of HK2 and PCK1 protein expression in LPS-stimulated BV2 cells following SIN pretreatment. (L) Expression analysis of stress-response genes (HspH1, Cryab, and Apod) following SIN intervention. Data are presented as the mean ± SD from at least three independent experiments. Statistical analyses were performed as described in the Methods. *P < 0.05, * *P < 0.01, * * *P < 0.001, and * * * *P < 0.0001 vs. the Control group; #*P* < 0.05, ##*P* < 0.01, ###*P* < 0.001, and ####*P* < 0.0001 vs. the LPS + Vehicle group.

The optimal LPS concentration for inducing inflammatory activation was subsequently determined. CCK-8 assays demonstrated that LPS exerted dose-dependent effects on BV2 cell viability. Concentrations of 125–250 ng/mL caused minimal cytotoxicity, whereas 500–1000 ng/mL significantly reduced cell viability (Figure 4B). In parallel, qRT-PCR analysis showed that LPS dose-dependently upregulated the pro-inflammatory markers *iNOS* and *IL-1β* while concomitantly downregulating *PCK1* expression (Figure 4C). Considering both inflammatory induction efficiency and cell viability, 250 ng/mL LPS was selected for subsequent modeling.

LPS exposure induced a pronounced inflammatory transcriptional response in BV2 microglia, as evidenced by elevated Nos2 and Il1b expression and decreased levels of Il10 and Mrc1. SIN pretreatment dose-dependently reversed these changes by suppressing the expression of pro-inflammatory genes while restoring the expression of anti-inflammatory markers (Figure 4D). Consistent with the transcriptional changes, cytokine analysis of culture supernatants showed that LPS markedly increased the secretion of IL-1β and TNF-α while reducing IL-10 and TGF-β levels. SIN treatment significantly attenuated IL-1β and TNF-α secretion and recovered the production of IL-10 and TGF-β, indicating a shift toward a more homeostatic microglial phenotype (Figure 4E). To further characterize the phenotypic changes induced by SIN, immunofluorescence staining was subsequently performed. LPS stimulation significantly increased iNOS expression and reduced CD206 expression compared with the control group, resulting in an elevated iNOS/CD206 ratio (Figure 4F). SIN pretreatment dose-dependently reversed the LPS-induced phenotypic changes, as evidenced by reduced iNOS immunofluorescence intensity and increased CD206 expression. Collectively, these findings suggest that SIN attenuates LPS-induced microglial inflammatory activation and restores microglial homeostatic phenotype.

#### 3.7.2 Sinomenine reduces oxidative stress and improves cellular stress responses

Consistent with its anti-inflammatory effects, SIN also attenuated oxidative stress in LPS-stimulated BV2 microglia. LPS markedly increased intracellular ROS accumulation, whereas SIN pretreatment dose-dependently limited ROS generation (Figures 4G,H). These results further support the ability of SIN to preserve cellular redox balance during inflammatory challenge.

Changes in cellular stress-response pathways were further examined by assessing the expression of representative stress-responsive genes. Compared with untreated cells, LPS stimulation markedly disrupted the expression of *HspH1*, *Cryab*, and *Apod*. SIN treatment reversed these alterations and restored stress-response gene expression patterns toward basal levels (Figure 4L), suggesting that SIN may enhance cellular adaptive responses under inflammatory stress conditions.

#### 3.7.3 Sinomenine modulates PCK1-associated metabolic remodeling and energy metabolism

To further investigate the metabolic alterations induced by LPS stimulation and the effects of SIN treatment, key genes involved in glucose metabolism were examined. Compared with untreated controls, LPS exposure induced pronounced metabolic remodeling in BV2 microglia, as reflected by the upregulation of *Hk2* and *Glut1* accompanied by a reduction in Pck1 expression relative to untreated cells (Figure 4J). These LPS-induced transcriptional alterations were progressively attenuated by SIN pretreatment, which reduced Hk2 and Glut1 expression while restoring Pck1 expression at the mRNA level. Western blot analysis showed that HK2 protein expression was significantly decreased in the LPS + SIN100 group compared with the LPS + Vehicle group, whereas PCK1 protein expression was significantly increased in the LPS + SIN100 group relative to the Control group (Figure 4K). These findings are consistent with the transcriptional results and support the involvement of PCK1-associated metabolic regulation.

Functional metabolic analyses further revealed alterations in cellular energy metabolism following LPS stimulation. Compared with control cells, LPS stimulation resulted in decreased intracellular ATP levels, indicating impaired energy production. SIN treatment significantly increased ATP production, particularly at higher concentrations, suggesting improved cellular energy metabolism after SIN administration. Although LPS stimulation increased lactate levels compared with the control group, this change did not reach statistical significance. SIN treatment, particularly at 100 μM, significantly reduced lactate levels compared with the LPS + Vehicle group, suggesting that SIN may modulate glucose metabolic handling under inflammatory conditions (Figure 4I). Furthermore, LPS stimulation resulted in a reduced NAD^+^/NADH ratio, indicating an altered intracellular redox state. However, SIN treatment did not significantly restore the NAD^+^/NADH ratio under the present experimental conditions, suggesting that the metabolic effects of SIN may not primarily depend on normalization of NAD^+^-dependent redox homeostasis (Figure 4I).

These findings indicate that SIN alleviates LPS-driven inflammatory, oxidative, and metabolic disturbances in BV2 microglia and promotes a shift toward a more balanced cellular state.

## 4 Discussion

Vascular dementia (VaD) is a prevalent neurodegenerative disorder and a leading cause of cognitive impairment; however, therapeutic interventions capable of altering disease progression remain limited. Increasing evidence indicates that VaD pathogenesis extends beyond cerebrovascular injury and involves complex interactions among neuroinflammation, oxidative stress, mitochondrial dysfunction, and metabolic disturbances (Raz et al., 2016; Hosoki et al., 2023; Lonkar et al., 2025; Shinjyo and Kita, 2021). Our integrative analyses highlighted extensive dysregulation of immune and metabolic signaling in VaD, with significant enrichment of pathways involved in leukocyte-mediated immunity and antigen processing and presentation, together with metabolic pathways associated with glycolysis, oxidative phosphorylation, and MAPK signaling. Integration with single-cell RNA sequencing further allowed us to identify the specific microglial populations associated with these immune-metabolic alterations. These findings suggest that VaD is characterized by coordinated immune activation and metabolic dysregulation rather than isolated vascular injury.

Consistently, emerging evidence from single-cell and spatial transcriptomic studies has revealed extensive immunometabolic remodeling across multiple neurological disorders. A common feature of these disorders is the transition of microglia toward disease-associated states accompanied by profound metabolic reprogramming (Hammond et al., 2019; Garg et al., 2025; Sadeghdoust et al., 2024).

Microglia are the primary immune cells of the central nervous system and play important roles in inflammatory regulation and tissue repair following vascular injury. Increasing evidence indicates that microglial activation is a dynamic process characterized by distinct transcriptional and functional states, which are closely associated with changes in the cellular microenvironment and metabolism (Tsuda et al., 2023; Li and Barres, 2018; Prinz et al., 2019; Colonna and Butovsky, 2017).

In our study, stress-response and antigen-presenting microglia were expanded in VaD, whereas phagocytic and MHC-II transitional microglia were reduced. These alterations suggest a redistribution of microglial functional states toward stress-adapted and immune-associated phenotypes during VaD progression. Similar patterns of microglial remodeling have also been reported in other central nervous system disorders (Garg et al., 2025; Hammond et al., 2019). Antigen-presenting microglia exhibited enhanced metabolic activity. Glycolysis, pyruvate metabolism, and lipid remodeling were all upregulated in this population. These metabolic changes likely support the increased bioenergetic and biosynthetic demands required for immune activation and antigen presentation. In contrast, stress-responsive microglia displayed elevated glycolytic and lactate-associated signatures together with reduced oxidative phosphorylation. This metabolic profile indicates a greater reliance on glycolysis and impaired mitochondrial function under chronic inflammatory conditions (Sadeghdoust et al., 2024). Consistent with these observations, mitochondrial dysfunction has been reported to drive metabolic reprogramming in microglia and alter glucose metabolism under inflammatory conditions (Fan et al., 2026). Together, these findings support the involvement of immunometabolic remodeling in regulating microglial functional states, with mitochondrial dysfunction and metabolic alterations contributing to microglial adaptation under pathological conditions.

Metabolic remodeling has emerged as an important feature of disease-associated microglia, with alterations in glucose metabolism, lipid metabolism, and mitochondrial function closely linked to inflammatory phenotypes and disease progression (Deczkowska et al., 2018; Qin et al., 2023; Tuddenham et al., 2024; Patel et al., 2022). Metabolic reprogramming is now recognized as a key regulator of immune cell function (Jha et al., 2015). Similar to peripheral immune cells, activated microglia undergo metabolic transitions that influence their functional phenotype (O'Neill and Pearce, 2016). Inflammatory activation is characterized by enhanced glycolysis, increased lactate production, mitochondrial dysfunction, and excessive reactive oxygen species (ROS) generation (Deczkowska et al., 2018; Baik et al., 2019; Li et al., 2022; Lauro and Limatola, 2020). These metabolic alterations may sustain inflammatory signaling and reinforce the activated microglial phenotypes. In our study, LPS stimulation induced pronounced metabolic remodeling in BV2 microglia. This response was characterized by increased GLUT1 and HK2 expression, altered lactate metabolism, reduced ATP production, and disruption of the NAD^+^/NADH balance. These metabolic abnormalities were accompanied by elevated expression of the pro-inflammatory mediators iNOS, TNF-α, and IL-1β. Similar metabolic alterations have been reported in activated microglia exposed to inflammatory stimuli (Orihuela et al., 2016; Xie et al., 2023). Together, these findings support the concept that glycolytic reprogramming is closely associated with inflammatory activation and disruption of cellular metabolic homeostasis.

Our findings further support the close interplay between metabolic dysfunction and inflammation. Metabolic stress promotes inflammatory activation, whereas persistent inflammation further exacerbates mitochondrial dysfunction and metabolic impairment (Lauro and Limatola, 2020). This reciprocal interaction may drive the progression of chronic neuroinflammation. Accordingly, preserving metabolic flexibility, rather than simply suppressing inflammatory mediators, may provide a more effective therapeutic approach.

PCK1 is traditionally recognized as a key metabolic enzyme that regulates gluconeogenesis and carbon flux (Grasmann et al., 2019). However, recent studies have expanded its functional role beyond glucose metabolism, linking PCK1 to cellular stress adaptation and metabolic plasticity (Yu et al., 2021; Verissimo et al., 2023). Altered PCK1 activity influences carbon flux, redox balance, and energy metabolism (Dalga et al., 2025). In our study, integrative analysis of transcriptomic data and SIN-related targets identified PCK1 as a candidate metabolic regulator associated with VaD pathology and the biological effects of SIN. Pathway analysis further connected PCK1-associated genes with glycolysis, mitochondrial function, and inflammatory signaling. Molecular docking also supported a favorable interaction between SIN and PCK1. Together, these findings suggest that PCK1-associated metabolic regulation may contribute to the effects of SIN on inflammatory and metabolic alterations in activated microglia.

Consistent with these computational predictions, LPS stimulation markedly reduced PCK1 expression in BV2 microglia, whereas sinomenine partially restored PCK1 expression at the transcriptional level. This change was accompanied by reduced expression of iNOS, TNF-α, and IL-1β, together with restoration of homeostatic microglial markers. Sinomenine reduced lactate levels under inflammatory conditions, increased ATP production, and influenced intracellular metabolic parameters, although the NAD^+^/NADH ratio was not significantly restored under the present experimental conditions. These metabolic improvements coincided with attenuation of inflammatory activation. Collectively, these results suggest that PCK1-associated metabolic regulation correlates with the maintenance of metabolic homeostasis and inflammatory responses. Although restoration of PCK1 expression was associated with the protective effects of sinomenine, whether PCK1 is required for these effects remains to be determined.

Sinomenine has long been recognized for its anti-inflammatory and neuroprotective activities (Hou et al., 2024; Li et al., 2025). Previous studies have primarily demonstrated these effects in experimental models of neurological diseases and have emphasized the ability of SIN to inhibit NF-κB signaling and reduce the production of pro-inflammatory cytokines (Hong et al., 2022; Hou et al., 2024). Whether SIN also regulates microglial metabolism has remained unclear. Our findings suggest that its protective actions extend beyond inflammatory suppression. Instead, SIN may exert protective effects by simultaneously modulating inflammatory signaling, energy metabolism, and cellular stress responses. These findings indicate that restoration of immunometabolic balance may represent an important mechanism underlying the effects of SIN on microglial homeostasis.

Oxidative stress is closely linked to microglial dysfunction during chronic neuroinflammation. Excessive ROS production can impair mitochondrial function and disrupt cellular metabolism, thereby sustaining inflammatory activation. SIN effectively reduced ROS accumulation in LPS-stimulated BV2 microglia. This reduction in oxidative stress was accompanied by improved metabolic parameters and altered stress-response pathways. Together, these results suggest that oxidative stress and metabolic remodeling are closely interconnected processes that contribute to microglial functional states (Keren-Shaul et al., 2017).

Interestingly, our study revealed that SIN modulated the microglial stress-response program. CRYAB and APOD expression was further enhanced, whereas HSPH1 levels recovered following LPS stimulation. These changes suggest that SIN may enhance adaptive stress responses while alleviating excessive cellular stress under inflammatory conditions. Adaptive stress pathways are increasingly recognized as key mechanisms for maintaining cellular homeostasis during inflammation. Molecular chaperones, antioxidant defenses, and proteostasis together preserve mitochondrial function and support cell survival under inflammatory conditions.

CRYAB (αB-crystallin), APOD, and HSPH1 participate in adaptive stress-response networks that are involved in cellular protection against inflammatory stress. CRYAB contributes to protein homeostasis and mitochondrial protection through its molecular chaperone activity (Clark and Muchowski, 2000), whereas APOD is involved in lipid homeostasis and resistance to oxidative damage (Dassati et al., 2014; Ganfornina et al., 2008; Sanchez and Ganfornina, 2021). HSPH1, a member of the HSP110 family, promotes protein refolding and protects cells against proteotoxic stress (Kampinga and Craig, 2010). Together, these stress-responsive pathways may enhance microglial resilience and support the maintenance of cellular homeostasis during inflammatory activation.

Our findings are consistent with the concept that enhancing cellular adaptation may represent an additional mechanism for limiting inflammatory injury (Medzhitov et al., 2012; Soares et al., 2017). SIN may therefore exert protective effects not only by suppressing inflammatory signaling but also by strengthening adaptive stress-response programs and enhancing cellular resilience. This coordinated regulation may contribute to the maintenance of microglial homeostasis under inflammatory conditions.

Current VaD management primarily focuses on vascular risk-factor control, whereas strategies directly targeting neuroinflammation and microglial dysfunction remain limited. The present data support the notion that modulating microglial bioenergetics may provide a promising therapeutic approach for VaD. Such an approach may complement existing treatment strategies by targeting inflammation-associated metabolic dysfunction. Rather than solely reducing inflammatory mediators, SIN may alleviate microglial dysfunction by correcting inflammation-associated metabolic disturbances and improving cellular adaptation. In association with PCK1-associated metabolic pathways, SIN was associated with reduced inflammatory activation, improved energy metabolism, and enhanced adaptive stress responses. Given that the neurological applications of SIN remain largely at the preclinical stage and that clinical evidence for vascular dementia is currently unavailable, our findings provide mechanistic support for the further preclinical development of SIN.

Several limitations should be acknowledged. Although PCK1 was identified as a candidate associated with SIN activity, its causal contribution requires further validation using genetic approaches, such as PCK1 knockdown or overexpression. In addition, the present findings are mainly based on BV2 microglial models and require confirmation in in vivo VaD models. As SIN-mediated neurological benefits remain at the preclinical stage, future pharmacokinetic and clinical studies are needed to determine its brain exposure and therapeutic potential in patients with vascular dementia.

## 5 Conclusion

In conclusion, this study reveals that sinomenine alleviates microglial inflammatory and metabolic disturbances through modulation of PCK1-associated metabolic regulation. Beyond its classical anti-inflammatory effects, sinomenine promotes metabolic flexibility and adaptive stress responses in activated microglia, thereby contributing to the maintenance of microglial homeostasis. Our findings broaden the current understanding of the pharmacological actions of sinomenine and identify microglial immunometabolic regulation as a potential therapeutic target for vascular dementia.

## Data Availability

The datasets analyzed during this study are publicly available in the Gene Expression Omnibus (GEO) repository under accession numbers GSE122063 and GSE282111 (https://www.ncbi.nlm.nih.gov/geo/).
